# Teaching evidence-based practice principles to prepare health professions students for an interprofessional learning experience

**DOI:** 10.5195/jmla.2017.179

**Published:** 2017-10-01

**Authors:** Nell Aronoff, Elizabeth Stellrecht, Amy G. Lyons, Michelle L. Zafron, Maryruth Glogowski, Jeremiah Grabowski, Patricia J. Ohtake

## Abstract

**Objective:**

The research assessed online learning modules designed to teach health professions students evidence-based practice (EBP) principles in an interprofessional context across two institutions.

**Methods:**

Students from nine health professions at two institutions were recruited to participate in this pilot project consisting of two online learning modules designed to prepare students for an in-person case-based interprofessional activity. Librarians and an instructional designer created two EBP modules. Students’ competence in EBP was assessed before and after the modules as well as after the in-person activity. Students evaluated the online learning modules and their impact on the students’ learning after the in-person session.

**Results:**

A total of 39 students from 8 health professions programs participated in the project. Average quiz scores for online EBP module 1 and module 2 were 83% and 76%, respectively. Following completion of the learning modules, adapted Fresno test of competence in EBP scores increased (*p*=0.001), indicating that the modules improved EBP skill competence. Student evaluations of the learning modules were positive. Students indicated that they acquired new information skills that contributed to their ability to develop a patient care plan and that they would use these information skills in their future clinical practice.

**Conclusions:**

Online EBP learning modules were effective in developing EBP knowledge and skills for health professions students. Using the same modules ensured that students from different health professions at different stages of their professional programs had consistent knowledge and enabled each student to fully engage in an interprofessional evidence-based activity. Student feedback indicated the modules were valued and beneficial.

## INTRODUCTION

Interprofessional education (IPE) has become an important element in the education of health professions students and is a component of many health care program accreditation standards [1]. The World Health Organization defines IPE as occurring “when students from two or more professions learn about, from and with each other to enable effective collaboration and improve health outcomes” [2].

In 2011, the Interprofessional Education Collaborative (IPEC), comprising a panel of experts from national associations of six health professions, published a report outlining the core competencies for interprofessional collaborative practice, the intended outcome of IPE. The four core competencies are values and ethics for interprofessional practice, roles and responsibilities, interprofessional communication, and teams and teamwork [3]. In 2016, the IPEC released an updated report that established interprofessional collaboration as the central domain and expanded the competencies to further the “Triple Aim,” a framework developed by the Institute for Healthcare Improvement to optimize health system performance. The three dimensions of the “Triple Aim” are improving the patient experience of care (including quality and satisfaction), improving the health of populations, and reducing the per capita cost of health care [4]. In the original article about the “Triple Aim,” the authors suggested that “physician-centric care” was a barrier to meeting these pursuits [5]. Interprofessional collaborative practice has been endorsed as an important transition in health care delivery that will further the “Triple Aim” by encouraging teamwork among health care providers [6–9].

Librarians are natural partners in IPE because they have experience engaging institutional stakeholders in different schools and departments. Despite this, few articles about library-related IPE activities appear in the published literature. Librarians’ involvement in IPE activities runs the gamut from providing physical space to spearheading campus initiatives [10, 11]. Librarians serve as instructors and facilitators in IPE courses, imparting information about health literacy and evidence-based practice (EBP) to students in different health professions [12–15]. They also partner with schools and faculty to lead the IPE charge at their institutions, actively participating as members of IPE committees and centers that incorporate IPE into curricula, plan conferences and programs, and write health literacy white papers [16, 17].

In the present study, librarians and faculty developed an interprofessional EBP education project using a flipped classroom format. Flipped classroom instruction “delivers lecture content to students at home through electronic means and uses class time for practical application activities” [18]. This kind of education blends online and in-person learning and is becoming more prevalent in the health sciences [19]. Studies implementing flipped classroom pedagogy suggest that this technique can promote critical thinking and encourage increased peer communication and teamwork [19, 20]. This educational strategy has been used to teach health sciences students information literacy and EBP concepts and is reported to improve basic library research skills [21, 22]. The purpose of this study is to examine the effectiveness of online learning modules for educating health professions students about EBP principles in preparation for an interprofessional in-person learning experience.

## METHODS

### Context

In 2014 and 2015, librarians and faculty members at the University at Buffalo and State University of New York (SUNY) Buffalo State in Buffalo, New York, embarked on a library-driven IPE pilot project: “Information Resources for Evidence-Based Interprofessional Health Care Decisions: Developing, Testing and Evaluating Library-Based Innovative Technology Enhanced Team Instruction Methods.” Two main components of this project were the development of (1) online learning modules and (2) an in-person interprofessional case-based activity utilizing the skills learned in the online modules. Librarians and faculty members from both institutions and all participating schools worked together to create and implement the pilot project.

### Participants

The authors recruited students from nine health professions programs to participate in the project. Medical, dental, pharmacy, nursing, occupational therapy, physical therapy, and social work students were recruited from the University at Buffalo. Speech language pathology and dietetics students were recruited from SUNY Buffalo State. To ensure that all students were at approximately equivalent stages of their education, the authors recruited students who were in the professional phase of their programs and had some clinical experience. Since this was a pilot feasibility project, the goal was to recruit seven students from each profession, which would allow the in-person learning experience to be offered twice in a technology-enhanced active learning classroom at SUNY Buffalo State. We obtained institutional review board approval from both institutions, and all participants gave written informed consent.

### Protocol

Upon enrollment in the project, students completed a pre-module assessment of their EBP knowledge and skills (adapted Fresno test of competence in evidence-based practice [AFT]). Next, students were provided with access to two online EBP learning modules that were hosted on the learning management system at each academic institution. Consistent with a flipped classroom educational strategy, online learning modules were chosen as the mode of delivery for the EBP content to permit students to access the information at their own rate, to assess their self-directed learning through short quizzes, and to relieve the logistical challenges of organizing face-to-face EBP instruction with students from multiple health professions programs at two institutions. Students were given one week to complete the online EBP learning modules. Following their completion of the modules, students completed the AFT a second time.

Students then engaged in a facilitated in-person interprofessional small group (approximately five students per group) learning experience. The students were provided with a fictitious case and asked to use their knowledge and skills to develop a plan of care supported by clinical evidence. Students completed the AFT a third time following the in-person learning experience.

Completion of the online EBP learning modules was required prior to participating in the in-person interprofessional activity. A small monetary incentive was provided to students upon their completion of the online learning modules, the in-person interprofessional activity, and all assessments and evaluations.

### Online learning module content and development

A team of two librarians and an instructional designer developed two hour-long online learning modules to familiarize students with EBP principles and basic literature searching techniques. Module 1 (“Introduction to Evidence-Based Practice”) introduced students to EBP and article appraisal, and module 2 (“Finding Evidence in PubMed”) provided PubMed instruction, including information about Medical Subject Headings (MeSH). The modules included content created by the librarians as well as online content available under Creative Commons licenses [23–27]. The goal of having all students complete the same online EBP learning modules was to ensure that all students had similar EBP knowledge and skills prior to participating in the in-person interprofessional activity.

### Assessments and evaluations

After completing each EBP module, students completed a quiz created by the module developers. The quiz for module 1 comprised five questions that assessed knowledge of EBP components; development of patient/population, intervention, comparison, and outcome (PICO) questions; study designs; and critical appraisal strategies. The quiz for module 2 comprised eight questions assessing students’ understanding of locating literature in PubMed using a variety of searching techniques, including searching with keywords, using MeSH terms, and limiting with PubMed filters.

The effectiveness of the online modules regarding students’ EBP knowledge and skills was assessed using the AFT, a valid and reliable case-based assessment tool that was initially designed for allied health professionals [28, 29]. Only the first five of the seven original questions from the published instrument were used, as the last two questions were not relevant to the scope of this project.

The test presented students with 3 different clinical scenarios. Students were asked to select one of the scenarios and use it to respond to questions on the following 5 subjects: creation of a PICO question, utilization of information resources, study design, search characteristics, and critical appraisal. The test was administered three times: before and after the online modules and after the in-person interprofessional learning activity. Each time the test was administered, 3 different clinical scenarios were used in an effort to reduce practice effects [29]. Therefore, a total of 9 different clinical scenarios from previously published AFT articles [28, 30–32] were used. The 5 AFT questions, which remained the same for each iteration, were worth 24 points apiece, for a total of 120 points. To reduce scoring bias, all tests (n=117) were placed in a random order and scored by a medical librarian who was not associated with the study and was blind to the order of presentation to the students.

Following the in-person interprofessional activity, students completed an author-developed anonymous evaluation of the modules. This evaluation used a 5-point Likert scale, with 1 representing “Strongly disagree” and 5 representing “Strongly agree.” Students were asked if they agreed or disagreed with the two following statements: new information was learned, and this information would impact their future practice. In addition, students were given the chance to provide feedback about the EBP and PubMed searching online modules.

### Data analysis

Module quiz scores were used to assess students’ EBP knowledge related to the online EBP learning modules. The total percentage quiz score for each module was calculated by summing the raw scores for each question and dividing by the total possible score to generate a percentage. Scores for the individual module quiz questions are expressed as percentages (95% confidence intervals [CI]). The AFT scores were used to assess the impact of the online EBP learning modules and the in-person interprofessional EBP learning experience on students’ EBP knowledge and skill development. AFT scores were calculated by summing the points earned for each question and are reported as means (95% CI). A one-way ANOVA for repeated measures was used to compare scores at the 3 time points (before and after the online EBP modules and after the in-person interprofessional EBP learning experience). Significant F-ratios were followed by Tukey’s honestly significant difference (HSD) post-hoc tests. Significance was set at *p*<0.05. Likert scale data from the student evaluations of the project are reported as medians (interquartile range [IQR]). Qualitative comments were reviewed, and common themes were identified by 2 investigators.

## RESULTS

Thirty-nine students from 8 health professions programs in the 2 institutions participated. Students were enrolled in dental medicine (n=5), dietetics (n=6), medicine (n=5), occupational therapy (n=6), pharmacy (n=4), physical therapy (n=6), social work (n=4), and speech language pathology (n=3). Due to a scheduling conflict, nursing students were unable to participate.

**Figure 1:**
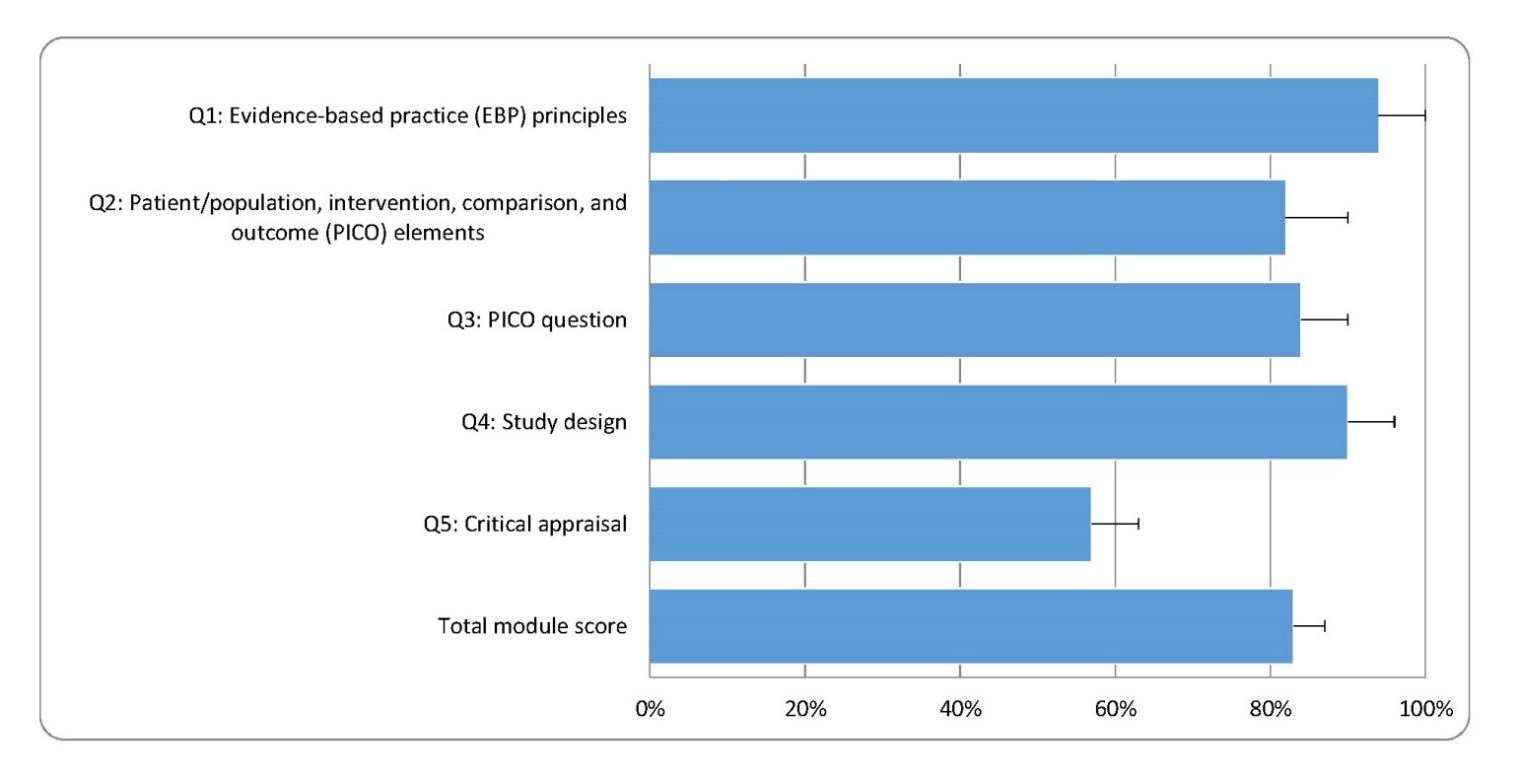
Total score and individual scores for module 1: “Introduction to Evidence-based Practice”^*^ ^*^ Data are presented as means (95% CI).

The average total quiz score for module 1, “Introduction to Evidence-Based Practice,” was 83% (95% CI (75%, 91%)) (Figure 1). Average student scores were above 80% for the quiz questions related to EBP principles (Q1), PICO question elements (Q2), PICO question development (Q3), and study designs (Q4). Student scores were lower on Q5, which assessed critical appraisal of an article.

**Figure 2:**
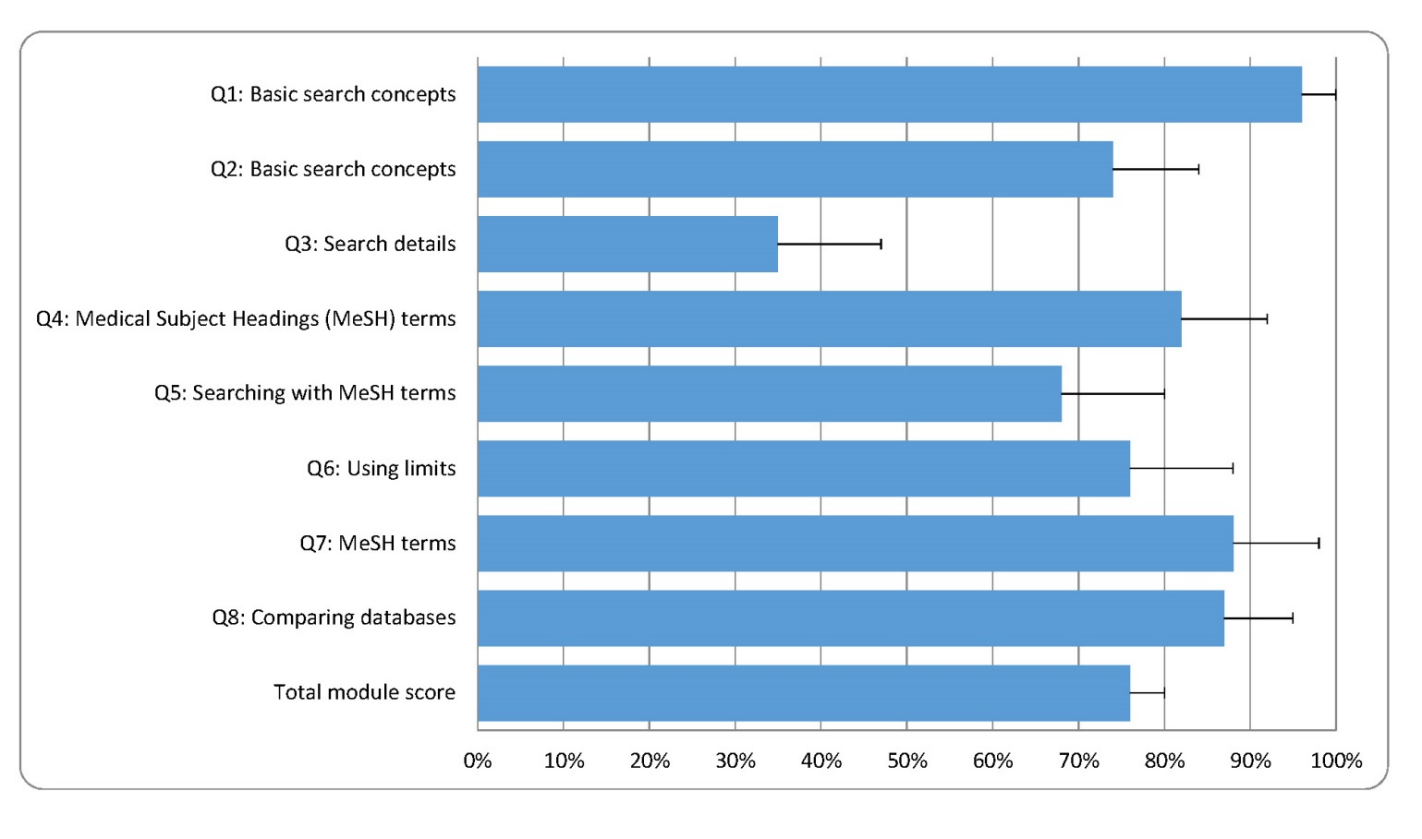
Total score and individual scores for module 2: “Finding Evidence in PubMed”^*^ ^*^ Data are presented as means (95% CI).

For module 2, “Finding Evidence in PubMed,” the average total quiz score was 76% (95% CI (72%, 80%)) (Figure 2). Students scored higher on questions pertaining to using basic search concepts (Q1 and Q2), identifying MeSH terms (Q4 and Q7), using limits (Q6), and comparing research databases (Q8). Students scored lower on questions pertaining to explaining search details (Q3) and searching for information using only MeSH terms (Q5). AFT scores changed significantly in response to the program (F(2,76)=8.417, *p*<0.001). The average AFT score before accessing the online learning modules was 64 (95% CI (59, 69)). After accessing the online EBP modules, the average AFT score increased to 73 (95% CI (67, 78); *p*=0.001). After the in-person interprofessional EBP learning experience, the average AFT score decreased to pre-module levels of 64 (95% CI (59, 69); *p*=0.003).

The majority of students indicated that they learned new information (median [IQR]; module 1: 5 [4–5]; module 2: 5 [4–5]). The students also felt that they would be able to use the information in their future practice (module 1: 5 [4–5]; module 2: 5 [4–5]). A total of 19 out of 39 students (49%) provided additional feedback. Many students (n=10) commented that they were appreciative of the content, especially in regard to the PubMed module.

## DISCUSSION

This study used online EBP learning modules to teach EBP knowledge and skills to health professions students to ensure a similar EBP knowledge baseline before an in-person interprofessional EBP learning experience. Recruited students had some clinical experience and an understanding of their own professions, but their exposure to EBP concepts was unknown. Therefore, students from different professional programs and different institutions engaged in the same online EBP learning modules. Creating a knowledge baseline enabled participants to start the in-person interprofessional learning experience with uniform EBP knowledge and skills, while still acting as content experts for their specific professions.

Results of this pilot study showed that the online learning modules were effective in teaching EBP principles and PubMed search skills. Similarly, a recent systematic review on EBP educational strategies for health professions students stated that “web based educational platforms have been demonstrated as an effective and desirable mechanism to deliver educational content to health professionals” [33]. According to Davis, online and face-to-face teaching yield comparable outcomes in improving students’ EBP knowledge [34]. In addition, first-year medical students who received online PubMed training performed as well as their predecessors who received in-person training. Furthermore, student satisfaction improved when the training moved online [35].

The online learning modules were designed with the following learning outcomes for student participants: describe the basic principles and steps of EBP, differentiate and define study designs, and successfully search for and locate evidence in PubMed. The PubMed database was selected for this project as it was freely accessible and covered a broad variety of health sciences disciplines. Although the modules were successful in teaching students how to identify types of study design, create focused clinical questions, and find evidence through basic searching in PubMed, students struggled with critical appraisal of research literature and use of MeSH to find evidence in PubMed, suggesting that the modules did not provide sufficient training to master these two skills. Consistent with this, student evaluations revealed that they were previously unfamiliar with MeSH. Based on the results of this pilot project, future iterations of the modules may contain more content about MeSH and critical appraisal of research articles.

The AFT was used to measure changes in students’ EBP knowledge and skills in response to the online EBP modules and the in-person interprofessional EBP learning experience. The AFT was originally designed for occupational therapists [28, 29] and was subsequently validated for physical therapists, speech language pathologists, social workers, registered dieticians, psychologists, and early educators [30, 36, 37]. After accessing the online learning modules, AFT scores increased by 14%, which is consistent with an educationally important change of 10%–15% [29]. Interestingly, however, the AFT scores returned to pre-online EBP learning module levels after the in-person EBP interprofessional learning experience, indicating that their EBP knowledge and skills were not retained. This pattern of an increase in AFT scores after EBP instruction, followed by a decrease in scores at follow-up, has been previously reported in studies of both occupational therapy students and physical therapist clinical instructors [38, 39].

While student scores on the modules suggest that there was room for improvement, student evaluations of the modules were very favorable. The evaluations demonstrated that students found the information to be useful, and students believed they would be able to apply the information learned from the modules in their future clinical practice. This was in keeping with an earlier study of IPE-centered modules with library skills content, in which a majority of students felt that library sessions were valuable and that the information they learned would help them in their programs and careers [40].

Student evaluations of both modules suggested that many students did not have previous exposure to the EBP information presented in the modules. Students’ comments were generally positive concerning both modules when given the chance to provide qualitative feedback. The most common theme to emerge was that students found the PubMed module in particular to be a useful introduction to or review of finding evidence in the literature. Overall, the students’ quantitative and qualitative evaluations implied that the module information was beneficial and well received.

There were limitations associated with this pilot feasibility study. First, there was no control group, and the sample size was small. Second, students volunteered five to ten hours of their time during the fall semester, which might have resulted in a self-selection bias. Typically, students who volunteer for additional opportunities outside of regular class time are high performers. Therefore, the results of this project might not be representative of all health professions students. Third, library science students pretested the modules. In retrospect, additional testing should have been conducted with students from health sciences professions programs to verify the clarity of module questions and students’ understanding. Finally, student performance on the AFT might have been negatively influenced by the clinical scenario selection. To prevent the possibility of a learning effect from repeated administration of the AFT [29], each version of the AFT provided students with three different previously published clinical scenarios [28, 30–32]. As not all professions were represented in the clinical scenarios on each version of the AFT, some students were forced to work with a clinical case that was out of their professional fields of expertise.

This project demonstrates that online learning modules can be used to teach EBP knowledge and skills in an IPE context. Providing consistent EBP knowledge and skills education to all health professions students afforded a similar baseline prior to participating in the in-person interprofessional learning experience, thereby integrating EBP into IPE. Furthermore, utilizing these skills contributes to patient care planning in clinical settings. As one student commented, “Evidence-based practice is key to helping create an effective treatment plan for clients and PubMed provides available research to create the treatment plan.”

Since the project concluded, librarians have been involved in planning and facilitating subsequent university-sponsored IPE activities. There has been discussion about using the modules for future IPE activities, as the results demonstrate that the project was feasible and could be scaled up to include a larger number of participants. Becoming involved in IPE learning activities enables librarians to contribute to the EBP knowledge and skills of health professions students and provides an opportunity for librarians to collaborate with faculty from various health professions programs and departments. Not only do projects like this serve to educate students, but they give librarians a seat at the interprofessional table.
